# Providers’ Non-Cigarette Tobacco Use Intervention Practices in Relation to Beliefs about Patients, Prioritization of and Skills for Intervention, and Referral Knowledge in Texas Healthcare Centers Providing Care to Persons with Behavioral Health Needs

**DOI:** 10.3390/ijerph192114269

**Published:** 2022-11-01

**Authors:** Midhat Z. Jafry, Sean M. Reuven, Maggie Britton, Tzuan A. Chen, Isabel Martinez Leal, Anastasia Rogova, Bryce Kyburz, Teresa Williams, Mayuri Patel, Lorraine R. Reitzel

**Affiliations:** 1 Department of Health Disparities Research, University of Texas MD Anderson Cancer Center, Houston, TX 77230, USA; 2 Department of Biology and Biochemistry, College of Natural Sciences & Mathematics, University of Houston, Science & Research Building 2, 3455 Cullen Blvd Room 342, Houston, TX 77204, USA; 3 Department of Psychological, Health & Learning Sciences, The University of Houston, 3657 Cullen Blvd Stephen Power Farish Hall, Houston, TX 77204, USA; 4 College of Medicine, University of Houston, 5055 Medical Circle, Houston, TX 77204, USA; 5 Health Research Institute, The University of Houston, 4349 Martin Luther King Blvd., Houston, TX 77204, USA; 6 Integral Care, Austin, TX 78704, USA; 7 Department of State Health Services, Tobacco Prevention and Control Branch, Austin, TX 78756, USA

**Keywords:** substance use disorders, mental health disorders, non-cigarette tobacco use, concurrent use, behavioral health providers, provider skills, referral knowledge, beliefs about patients

## Abstract

Rates of non-cigarette (colloquially, other) tobacco use is elevated among adults with behavioral health conditions. Little is known about whether behavioral health providers are using brief interventions, including the evidence-based 5As (Ask, Advise, Assess, Assist, and Arrange) for other tobacco use, or what provider factors may be associated with use of these interventions. The current study redressed this gap. Overall, 86 providers in Texas (9 Federally Qualified Health Centers, 16 Local Mental Health Authorities (LMHAs) that provide a broad range of mental and behavioral health services, 6 substance use treatment programs in LMHAs, and 55 stand-alone substance use treatment programs) took a survey assessing their beliefs regarding (1) patients’ concerns about other tobacco use; (2) their desire to quit; (3) importance of intervening on other tobacco use with cessation counseling; (4) perceived skills to intervene; (5) knowledge of referral options for treatment. Logistic regression analyses were conducted to determine the association between each factor and use of the 5As. Results showed that 70.9% of providers asked patients about other tobacco use status, 65.1% advised them to quit, 59.3% assessed quit interest, 54.7% assisted with a quit attempt, and 31.4% arranged a follow-up. Providers who believed patients were concerned about other tobacco use, recognized the importance of offering other tobacco use cessation counseling, believed they had the necessary skills to treat other tobacco use, and possessed knowledge of referral options, respectively, were more likely to deliver the 5As (*ps* < 0.05). Results add to a limited literature on provider intervention practices for other tobacco use in settings where behavioral health care is provided, highlighting the significance of provider beliefs, perceived skills, and referral knowledge to care delivery. Findings reveal opportunities to increase delivery of the 5As for other tobacco use to behavioral health patients and suggest provider factors that could be targeted to build this capacity.

## 1. Introduction

The health impacts of conventional cigarette smoking are well known; however, the use of non-cigarette tobacco products (namely, cigars, smokeless tobacco, and pipes, water pipes, or hookahs) has similarly long-term deleterious effects on health, including an increased risk of cancer, cardiovascular disease, and respiratory conditions [1]. The health risks of non-cigarette tobacco use (hereafter, other tobacco use) are concerning; studies have cited, for example, that hookah poses an equal or even greater risk to an individual’s health than cigarette smoking [2,3], cigar smoke contains more cancer-causing substances than cigarette smoke [4], and use of smokeless tobacco products is linked to oral cancer and cardiovascular disease [5]. A national survey in the U.S. indicated that 3.7% of American adults aged 18 years or older (9.1 million) used e-cigarettes, 3.5% (8.6 million) used cigars, 2.3% (5.7 million) used smokeless tobacco, and 1.1% (2.6 million) used pipes, water pipes, or hookahs in 2020 [6]. Furthermore, whereas the prevalence of conventional cigarette use declined in the U.S. from 2019 to 2020, rates of some forms of other tobacco use have not [6]. Consequently, more work is needed in the U.S. that specifically targets reductions in other tobacco use to achieve public health gains like those seen with conventional cigarette smoking. 

Although rates of other tobacco product use at the national level may seem minor relative to conventional cigarette use, cited at 12.5% in 2020 [6], these overall estimates hide special groups who use other tobacco products at comparatively higher rates. For example, rates of use in the U.S. among those experiencing regular depressive or anxiety symptoms ranged from 7.1–8.3% for e-cigarettes (versus 3.7% for adults overall), 3.7–4.1% for cigars (versus 3.5%), 2.1–2.6% for smokeless tobacco (versus 2.3%), and 1.8–2.8% for pipes, water pipes, or hookahs (versus 1.1%) in 2020 [6]. This disparity is even more marked when all forms of behavioral health needs (i.e., non-nicotine substance use disorder and/or mental health diagnoses) are taken into account. For example, a 2016 report revealed that 30.5% of adults receiving care in substance-use treatment centers used e-cigarettes [7]. Likewise, a study published in 2017 using 2013 and 2014 data from the National Survey on Drug Use and Health reported that whereas adults with no chronic health conditions smoked cigars at a rate of 9.1–9.4%, adults with non-nicotine substance use disorders smoked cigars at rates between 25.5–28.0% (weighted averages) [8]. This pattern was similarly evident in smokeless tobacco rates, with 11.4–11.7% of adults with substance use conditions reporting use of smokeless tobacco, a rate almost triple that of those with no chronic health conditions (4.2–4.3%) [8]. Given the health risks other tobacco use poses, adults with behavioral health needs represent a critical priority group for future tobacco control efforts.

Despite extensive literature on provider interventions for cigarette smoking cessation within behavioral health (mental health and/or substance use) treatment centers, which largely indicate a need to further enhance services offered [9,10,11], there are few reports specifically highlighting the provision of treatment to adults receiving care in these settings who use other tobacco products. National guidelines recommend the provision of evidence-based interventions, specifically the “5As” intervention model for all tobacco products: Ask about other tobacco use, Advise other tobacco users to quit, Assess other tobacco users’ readiness to quit, Assist other tobacco users through provision of cessation resources, and Arrange a follow-up with patients to check on quit attempt progress or to explore interest in quitting anew [12]. Despite the 5As model being widely recognized as the gold standard, data suggest that it is inconsistently provided to patients in behavioral health treatment settings [13]. Specifically, national data from 2016 indicated that only 48.9% of mental health treatment centers and 64.0% of substance use treatment centers screened patients for any tobacco use (including but not limited to conventional cigarettes) [13]. Moreover, only 27.6% of mental health treatment centers and 47.4% of substance use treatment centers in Texas provided tobacco cessation counseling [13]. Nicotine replacement therapy and medication provision were even less common in these settings [13]. Although these screening and treatment estimates were not reported specific to other tobacco use, there may be little reason to believe that provider intervention on comparatively less prevalent forms of tobacco use would exceed that of their intervention on the more common conventional cigarette smoking, particularly when the evidence-based intervention recommendations for cessation are equivalent. Moreover, while efforts have been recently undertaken to promote interventions for e-cigarette use specifically, these efforts have largely been limited to adolescents and young adults and are typically provided within settings other than where patients with behavioral healthcare needs are commonly treated e.g., [14,15]. Thus, these data underscore the need for more consistent implementation of the 5As in settings where patients with behavioral health needs receive care. This includes but is not limited to behavioral healthcare centers, considering that treatment guidelines recommend that tobacco use should be addressed by providers at every clinical touchpoint [12], which may be even more critical for adults with behavioral health needs.

Gaps in provision of treatment for other tobacco product use highlight an opportunity to build the capacity of treatment providers in settings providing behavioral health care to actively address their patients’ other tobacco use. As frontline treatment professionals, provider attitudes can significantly shape patients’ perceptions of the harms of other tobacco use and shape patients’ motivation to undertake a quit attempt [16]. Findings from a study on e-cigarette use suggested that provider misconceptions, such as the unfounded belief that patients are not interested in quitting other tobacco use, may impede their delivery of cessation treatment [17]. Furthermore, providers are often not specifically trained for treating tobacco use of any kind [18,19]. However, both attitudes toward cessation and knowledge of national guidelines have been associated with providers’ implementation of the 5As intervention in substance use treatment settings [20]. Likewise, data suggest that enhancing provider preparedness to treat other tobacco use, in this case smokeless tobacco, increases smokeless tobacco use quit rates [21]. However, this intervention was conducted among dental providers; very little research has been conducted regarding provider preparedness interventions and the provision of evidence-based interventions for other tobacco use in behavioral health treatment settings. However, the single recent study examining this within substance use treatment centers in Texas, U.S., indicated that a comprehensive tobacco-free workplace program that included intervention training led to increases in providers’ use of the 5As for other tobacco use with patients [22]. To the best of our knowledge, however, no prior work has reported on the use of the 5As by providers in myriad treatment settings that provide care for individuals with diverse behavioral healthcare needs, nor their association with provider factors such as their beliefs about patients’ concerns about use or interest in quitting or their preparedness to intervene to promote other tobacco use abstinence.

Given the limitations stated above, the current study expands the extant literature to more thoroughly understand providers’ use of brief intervention practices, including the 5As, for other tobacco use specifically, and within several treatment settings where patients with behavioral health needs receive care in Texas. Moreover, potential relationships between several provider factors (specifically, beliefs about patients’ concerns and desire to quit other tobacco use, perceived importance of providing treatment for other tobacco use, perceived skills to treat other tobacco use, and knowledge of referral options for other tobacco use care) and use of brief interventions for other tobacco use were explored. Results of this work may support the need to enhance the provision of care for other tobacco use in the settings where adults with behavioral health needs are commonly seen, while highlighting potential provider factors as targets for screening and intervention capacity building. Ultimately, these initial efforts may contribute to an enhanced public health focus on reducing the morbidity and mortality experienced from other tobacco use in the U.S., particularly in population subgroups with elevated use. 

## 2. Materials and Methods

### 2.1. Targeted Healthcare Centers

Data were collected as part of a contracted statewide needs assessment of Federally Qualified Health Centers (FQHCs), Local Mental Health Authorities (LMHAs), dedicated substance use treatment programs within LMHAs, and stand-alone substance use treatment programs that was designed to understand organizational practices regarding addressing patients’ tobacco use. These stakeholders were targets for the needs assessment due to their reach to patients with behavioral health care needs across the state of Texas. The needs assessment was extremely comprehensive and meant to guide future work across the state to meet tobacco control needs with evidence-based resources. Results of the needs assessment were not practical or feasible to present within a single scientific publication; the current study represents a secondary analysis from the overall report provided to the funder. Data collection began in April of 2021 and concluded in December of 2021.

### 2.2. Survey Methods and Response Rates

The study did not meet the definition of human subject research per the University of Houston’s IRB. A cover letter describing the purpose of the study and key elements of informed consent preceded a Qualtrics survey link. Because this was intended to be a needs assessment to guide future services provided within the state, the academic team in consultation with the community partners (co-authors on this report), opted on breadth versus depth of data collection. Therefore, only 1 survey was requested per physical location. The email and cover letter to the survey asked that the center send the survey to “to an appropriate person who would be keenly aware of tobacco service provision within your organization’s substance use/chemical dependency programs” for completion. The survey required identifying information including about the healthcare center, the respondent’s name, job title, and official email address (if applicable (some smaller centers do not provide employees email addresses)) for verification purposes, and whether they were a direct service provider or a general employee with no direct patient care provision. As the center selected survey respondents, they comprised a variety of center personnel, some of whom were direct service providers and other who were not. Direct service providers completing the survey were asked additional items about their practices that non-service providers were not. Remuneration for participation was a $20 Amazon gift card for fully or mostly (i.e., ~75%) completing the survey; respondents could “opt in” for remuneration through a prompt that allowed them to comply with employer rules regarding compensation and an email address where they wished to receive the gift card.

Recruitment of centers included direct email or postal mail solicitation (when email information was not available online). Several professional groups and individuals also assisted with recruitment by sending the study team their member lists or directly emailing their member listservs with information about the study. Recruitment was also aided by presentations in regular meetings of the Tobacco Prevention and Control Coalitions, regional coordinator meetings, and Community Resource Coordination Groups across the state.

The study team solicited participation from FQHCs (*n* = 57); all LMHAs, which provide a broad array of care for mental and behavioral health problems (*n* = 39; hereafter, global LMHAs to reflect their wide diagnostic scope); identified substance use treatment programs in LMHAs, which have a relatively more narrow focus on providing care for one or more substance use disorders and distinct regulatory requirements and management teams (*n* = 89); identified stand-alone substance use treatment centers (*n* = 458) in Texas. We sought 1 survey per physical healthcare location. After the elimination of duplicate surveys (*n* = 10, representing 5 centers), response rates were as follows: 43.9% of FQHCs (*n* = 25/57), 76.9% of global LMHAs (*n* = 30/39), 15.7% of substance use treatment programs within LMHAs (*n* = 14/89), and 14.4% of stand-alone substance use treatment centers (*n* = 66/458). The current study, focused on treatment practices, only used the surveys that were completed by direct service providers. Thus, the analyzable sample was: 9 FQHCs, 16 global LMHAs, 6 substance use treatment programs in LMHAs, and 55 stand-alone substance use treatment centers. 

### 2.3. Measures

#### 2.3.1. Healthcare Center Characteristics

Descriptive healthcare center characteristics included: (1) the number of unique patients seen annually (later categorized based on sample distribution as 50–200; 201–1000; and >1000); (2) the number of full-time employees (later dichotomized based on sample distribution as 1–50 vs. >50); (3) whether the center employed a person trained as a Certified Tobacco Treatment Specialist (CTTS; yes vs. no/I don’t know); (4) whether the center had a comprehensive tobacco-free workplace policy, defined as disallowing tobacco use inside buildings and anywhere on the property (yes vs. no). 

#### 2.3.2. Healthcare Center Provider Beliefs about Patients, Perceived Importance of and Skills for Treating Non-cigarette Tobacco Use, and Referral Knowledge

Providers were asked to rate their level of agreement with the following items: (1) “My patients are concerned about other (non-cigarette) tobacco use,” (providers’ belief about patients’ use concerns); (2) “My patients who use other (non-cigarette) tobacco products want to quit,” (providers’ belief about patients’ desire to quit); (3) “Non-cigarette tobacco use cessation counseling is an important part of my job” (perceived importance of providing cessation counseling); (4) “I have the required skills to help my patients quit other (non-cigarette) tobacco use (e.g., vaping products, smokeless tobacco, etc.),” (perceived skills to treat); (5) “I know where to refer patients for help with quitting other (non-cigarette) tobacco use (e.g., vaping products, smokeless tobacco, etc.),” (has referral knowledge). Each item was rated along a 5-point Likert scale ranging from strongly disagree to strongly agree. For analytic purposes, endorsements of strongly disagree, somewhat disagree/disagree, or neither agree nor disagree were compared with agree or strongly agree for each item.

#### 2.3.3. Healthcare Center Provider Non-Cigarette Tobacco Use Intervention Practices

Providers answered items regarding their non-cigarette tobacco use intervention practices, including their use of the 5As: In the last month, how frequently did you (1) “Ask your patients whether they used other forms of tobacco (e.g., vaping products, smokeless tobacco, etc.)?”; (2) “Advise your patients to quit other forms of tobacco?”; (3) “Assess your patients’ interest in making a non-cigarette tobacco use quit attempt?”; (4) “Assist your patients with quitting non-cigarette tobacco use via referral to the Texas Tobacco Quitline, via on-site referrals (e.g., to an internal prescriber, to a group cessation program), via off-site referrals (e.g., to an external prescriber, to a community group cessation program), or via direct intervention (counseling, medication)?”; (5) “Encourage your patients who use tobacco products other than cigarettes to reduce non-cigarette tobacco use, if they stated they could not quit?”; (6) “Arrange a follow-up contact at which progress with quitting non-cigarette tobacco use was discussed?” Each item was rated along a 5-point Likert scale ranging from never to always. For analytic purposes, endorsements of never, sometimes, or about half the time were compared with endorsements of most of the time or always on each item.

### 2.4. Data Analyses

Data were reported with descriptive statistics. Logistic regression analyses were conducted to assess the associations between the dependent variables (respondent’s other tobacco use intervention practices with patients (i.e., 5As and encouragement to reduce use)) and the independent variables (providers’ beliefs about patients, perceived skills, and referral knowledge). Healthcare center type was included as a covariate in the logistic regression analyses. Significance level was designated at *p* < 0.05. All analyses were conducted using SAS version 9.4.

## 3. Results

### 3.1. Healthcare Center Characteristics, including Overall Provider Information

Table 1 summarizes the characteristics of the healthcare centers participating in the current report, providers’ beliefs about patients, providers’ beliefs about treatment capability, and their intervention practices for non-cigarette tobacco use with patients. 

### 3.2. Providers’ Beliefs about Patients’ Other Tobacco Use in Relation to Intervention Practices

Logistic regression analyses, adjusted for healthcare center type, revealed that providers who believed patients were concerned about their other tobacco use had greater odds of Arranging a follow-up appointment to discuss quit progress relative to providers who did not agree that their patients were concerned about their other tobacco use. Providers’ beliefs about their patients’ desire to quit other tobacco use was not significantly related to their intervention practices, *including* the practice of encouraging a reduction in use for those who were uninterested in quitting (Table 2). 

### 3.3. Provider’s Perceptions of the Importance of Treating Non-Cigarette Tobacco Use, Skills to do so, and Referral Knowledge in Relation to Intervention Practices

Results indicated that providers who acknowledged the importance of offering tobacco use cessation counseling to patients for other tobacco use had greater odds of every intervention behavior assessed. Specifically, relative to their provider counterparts who did not perceive that other tobacco use counseling was an important part of their job, they were more likely to Ask their patients about non-cigarette tobacco use, Advise them to quit, Assess their interest in quitting, Assist with the quit attempt through direct intervention or referral, encourage a reduction in use if the patient stated they could not quit, and Arrange a follow-up appointment to discuss progress (Table 2).

Providers who believed they had the required skills to help their patients quit other tobacco use had greater odds of Asking their patients about other tobacco use, Assessing their interest in quitting other tobacco use, and Arranging a follow-up appointment to discuss quit progress relative to providers who perceived themselves as less skilled (Table 2).

Finally, providers who had knowledge of where to refer clients for help with other tobacco use cessation had greater odds of Assessing patients’ interest in quitting other tobacco use and in Arranging a follow-up appointment to discuss quit progress relative to providers with less referral knowledge (Table 2).

## 4. Discussion

The U.S. national guidelines recommend the provision of the evidence-based 5As for other tobacco use in adults with behavioral health needs [12]. This study highlights gaps in adherence to the 5As for non-cigarette (aka other) tobacco use in health care settings that serve this group. Ideally, all patients should be screened for other tobacco use in addition to conventional cigarette smoking at each clinical encounter, and those who endorse use should be provided with brief intervention and/or referral for care in 100% of cases. However, findings reveal only a third of providers reported arranging a cessation follow-up appointment and less than half encouraged reducing other tobacco use when the patient expressed an inability to quit. The remaining 5A components, while used at relatively higher rates, were each practiced by less than 75% of reporting providers. These findings align with those of a recent study conducted in substance use treatment centers in Texas that found low rates of providers’ use of the 5As for other tobacco use (e.g., 39.1% for arranging a follow-up to 67.7% for assessing interest in quitting) prior to a workplace intervention [22]. The failure to intervene on other tobacco use in all clinical encounters represents a missed opportunity to reduce the potential impact of tobacco use among a group overburdened by its health effects. Thus, findings reflect a need for greater prioritization of brief intervention provision for other tobacco use across settings where people with behavioral health needs receive care.

Findings of this study also reveal prevailing misconceptions among providers that may impact their care delivery for other tobacco use [17]. Specifically, less than half reported believing that their patients were concerned about their other tobacco use and possessed a desire to quit. This belief is counter to numerous studies finding that people with behavioral health conditions are interested in quitting tobacco use [17,23,24] and can successfully quit tobacco use [25]. Although most of this research has been conducted on conventional cigarette smoking, little to no evidence exists to suggest that it is not generalizable to other tobacco products that also convey health hazards from use. In this study, providers who believed that their patients were concerned about their other use were almost 2.8 times more likely to arrange a follow-up appointment to assess quit progress (or reassess interest in quitting) than their counterparts. These findings suggest a need for increased provider training that includes corrective information about patients’ concerns about their other tobacco use, the nature of quit motivation as something that can wax and wane over time, the provider’s key role in potentially shaping patient’s concerns about other tobacco use, and the ways in which making assumptions about patients’ needs can jeopardize their engagement in treatment and, ultimately, their health. Additionally, interventions aimed at reshaping provider beliefs may also benefit from specific attention on spiritual thinking, professional affiliations, and the provider’s sex, which have been cited as significant explanatory factors for the variance in beliefs on addiction among providers in addiction settings [26]. In conjunction with training, empowering patients to communicate their concerns and perceptions about their other tobacco use may further facilitate the reduction of provider misconceptions. This might be encouraged through the strategic placement of health promotion materials (e.g., posters within treatment rooms encouraging the patient to talk to their provider about their tobacco use). 

Relative to potential inaccuracies in providers’ beliefs, there were also provider strengths in these settings. Specifically, most providers perceived cessation counseling as important, believed they possessed the necessary skills to treat other tobacco use, and reported having knowledge of referral options for other tobacco use care. Notably, providers who perceived offering cessation counseling as important were more likely to practice the 5As than their counterparts. Most significantly, these providers were 5.9 times more likely to ask about other tobacco use, 7.2 times more likely to assist in a quit attempt, and 9.8 times more likely to recommend a reduction in use for those who did not want to quit than their counterparts. These findings complement other work linking behavioral health treatment providers’ attitudes toward smoking cessation with their practice of the 5As [20] and extend them to other tobacco use. Likewise, providers who believed they possessed skills to treat other tobacco use were 3.5 times more likely to ask patients about tobacco use and assess interest in quitting and 10.5 times more likely to arrange a follow-up than their less-skilled counterparts. Similarly, knowledge of referral options was linked with 3.5 times the odds of assessing patients’ interest in quitting and 4 times the odds of arranging a follow-up appointment. Interestingly, providers’ knowledge of referral options was not associated with assisting patients to quit, suggesting that knowledge by itself may not necessarily translate to action taken. Referrals, such as those to the Texas Tobacco Quitline, can be completed with very minimal time and effort on behalf of the provider. Future research should redress this knowledge to practice gap to support patients in quitting even when internal resources are not available. 

Together, these results suggest the importance of providers’ perceptions of treatment capability in their intervention on other tobacco use with patients and provide important areas to target in training to build providers’ capacity for care provision. Moreover, initial evidence suggests that education can change providers’ misconceptions about patients’ other tobacco use and significantly increase providers’ use of the 5As for other tobacco use, at least within substance use treatment settings [22]. Thus, healthcare centers providing care to patients with behavioral health needs might consider training sessions on these topics that are required for providers and offering them at regular intervals (annually) or events (new employee trainings), which will reduce the impact of provider turnover. Additionally, centers can promote attendance to freely available online education that often have the benefit of providing continuing education credits (e.g., those offered by the American Lung Association or the Smoking Cessation Leadership Center). Additionally, a “train-the-trainer” program can be considered to build and sustain capacity for the treatment of other tobacco use through building a community of practice within the center [27,28]. Importantly, at least one prior study has suggested that training opportunities may be welcomed by providers; a study on vaping and e-cigarettes revealed that providers perceived a need for increased clinical skills training especially on treatment options for other tobacco use [29]. However, it is important to keep in mind that excessive training requirements in behavioral health treatment settings have been associated with training fatigue [24]. More work may be necessary to inform how to strike an ideal balance of meeting providers’ needs, building capacity for patient intervention, and avoiding training fatigue. Finally, compiling and promoting providers’ use of local referrals for other tobacco use treatment, as well as national or state resources such as tobacco quit-lines and evidence-based cessation smartphone apps can be helpful in settings where direct counseling is not feasible. 

Despite addressing the largely unexplored area of other tobacco use treatment and its association with provider-level factors in settings that serve adults with behavioral health needs, the present study has several limitations. Specifically, the cross-sectional design prevents any causation from being inferred. Furthermore, all responses were self-reported, thus may have been influenced by recall bias, social desirability, and other factors. The surveys also exclusively assessed use of the 5As intervention and making a recommendation to reduce other tobacco use for those not ready to quit; thus, findings may not be representative of associations with other evidence-based interventions. Additionally, this study grouped all non-cigarette forms of tobacco into one category of “other tobacco use”; however, there may be differences within subtypes of other tobacco use and their relations with provider factors that were not able to be assessed in this study. For example, providers could believe that patients were concerned about their smokeless tobacco use but not their e-cigarette use. Moreover, the external validity of this research is necessarily limited by its design and aim; provider responses may not account for other provider perspectives within the same treatment center, or similar healthcare centers within Texas, or centers outside Texas. The relatively small sample size further supports that results may not be generalizable beyond the present sample of centers. However, it provides information that can guide future research or needs assessments within other states and settings. Further, the survey did not query specific provider characteristics, such as length of employment or years of experience in the field. The assessment of variation in provider beliefs and practices within the same healthcare location is also important to address in future work, as is their potential origin in individual provider characteristics. Likewise, examining these factors by healthcare location type is also important to adapt evidence-based workplace interventions to build capacity for non-cigarette tobacco care provision to the center’s inner context. Given these limitations, future steps in advancing tobacco cessation research should assess these factors longitudinally on a national scale and expand the scope of data collection methods (i.e., more providers within centers, incorporating qualitative methods to complement quantitative assessments), and the number of participating centers.

## 5. Conclusions

In summary, the present study reflects gaps in cessation care provision for non-cigarette tobacco use in settings where adults with behavioral health needs are routinely seen for healthcare. Results highlighting links between provider’s beliefs about patients’ other tobacco use and their self-perceptions of preparedness to provide interventions to patients with their provision of brief interventions for other tobacco use, including the 5As. These results suggest a need for provider training to correct misperceptions and to build treatment capacity in the participating healthcare centers. The present study is an important contribution to the literature given its expansion beyond exploration of healthcare practices regarding patients’ conventional cigarette dependency to specifically address practices regarding patients’ use and dependency on noncigarette tobacco products, an area that has been heretofore understudied and/or underreported, but is important given disparate use rates among adults with behavioral health needs.

## Figures and Tables

**Table 1:** Healthcare Center Characteristics; Providers’ Beliefs, Knowledge, Perceived Capacity to Treat Non-cigarette Tobacco Use; Non-cigarette Tobacco Use Intervention Practices (*n* = 86 healthcare centers).

Variables of Interest	All Centers
	% [*n*]
Healthcare Center Characteristics	
Center type	
Federally Qualified Health Center	10.47 [9]
Substance use program within LMHA	6.98 [6]
Global LMHA	18.60 [16]
Stand-alone substance use treatment center	63.95 [55]
# of unique patients seen annually	
50–200	32.10 [26]
201–1000	44.44 [36]
>1000	23.46 [19]
# of full-time employees	
1–50	61.45 [51]
>50	38.55 [32]
Provider’s Beliefs about Patients and Perceptions of Treatment Capability	
Providers’ beliefs about patients’ concerns regarding other tobacco use	37.21 [32]
Provider’s belief about patient’s desire to quit other tobacco use	44.19 [38]
Perceived importance of providing other tobacco use cessation counseling ^§^	67.07 [55]
Perceived skills to treat patient’s other tobacco use	66.28 [57]
Has knowledge of other tobacco use cessation referral options	75.58 [65]
Providers’ Non-Cigarette Tobacco Use Intervention Practices with Patients	
Asks patients about other tobacco use	70.93 [61]
Advises patients to quit other tobacco use	65.12 [56]
Assesses patient interest in quitting other tobacco use	59.30 [51]
Assists other tobacco use quit attempt	54.65 [47]
Encourages reducing other tobacco use if patient stated they could not quit	44.19 [38]
Arranges a tobacco use cessation follow-up	31.40 [27]

Note. LMHA = Local Mental Health Authority; ^§^ 4 missing values, # = number.

**Table 2:** Providers’ Non-Cigarette Tobacco Use Intervention Practices with Patients Relative to Their Beliefs about Patients’ Other Tobacco Use and Their Perceptions of Their Own Treatment Capability (*n* = 86 healthcare centers).

Provider’s Beliefs about Patients and Perceptions of Their Own Treatment Capability	Ask about Use	Advise to Quit	Assess Quit Interest	Assist Quit Attempt	Encourage Use Reduction	Arrange Follow-Up
	OR [95% CI]					
Providers’ belief about patients’ concerns regarding other tobacco use	1.313 [0.489, 3.527]	2.662 [0.969, 7.316]	1.878 [0.736, 4.789]	1.473 [0.591, 3.673]	2.393 [0.939, 6.098]	2.761 [1.044, 7.299]
Provider’s belief about patient’s desire to quit other tobacco use	1.280 [0.495, 3.311]	1.667 [0.661, 4.206]	1.366 [0.560, 3.330]	0.947 [0.393, 2.284]	1.738 [0.704, 4.292]	2.156 [0.833, 5.579]
Perceived importance of providing other tobacco use cessation counseling ^§^	5.945 [2.010, 17.579]	4.255 [1.528, 11.844]	3.816 [1.412, 10.307]	7.211 [2.440, 21.307]	9.783 [2.731, 35.046]	5.666 [1.495, 21.474]
Perceived skills to treat other tobacco use	3.506 [1.262, 9.739]	2.138 [0.816, 5.605]	3.460 [1.299, 9.217]	1.732 [0.674, 4.446]	2.483 [0.903, 6.833]	10.549 [2.235, 49.800]
Has knowledge of other tobacco use cessation referral options	1.798 [0.623, 5.186]	2.165 [0.773, 6.064]	3.415 [1.163, 10.03]	2.885 [0.971, 8.573]	1.590 [0.542, 4.662]	4.037 [1.028, 15.853]

Notes. Analyses controlled for center type; OR = Odds Ratio; CI = Confidence Interval. ^§^ 4 missing values. Reference category for column 1 = strongly disagree, somewhat disagree/disagree, or neither agree nor disagree. Reference category for columns 2–7 = never, sometimes, or about half the time.

## Data Availability

The data presented in this study are available upon request from the corresponding author. The data are not publicly available due to funder restrictions and because outcome papers are still being reported from the dataset.
